# Fluoroquinolone Residues in Piglet Viscera and Their Impact on Intestinal Microbiota Resistance: A One Health Approach

**DOI:** 10.3390/microorganisms13061389

**Published:** 2025-06-14

**Authors:** Olga Cardoso, Maria Manuel Donato, Sara Carolina Henriques, Fernando Ramos

**Affiliations:** 1 Chemical Engineering and Renewable Resources for Sustainability (CERES), Department of Chemical Engineering, Faculdade de Farmácia, Universidade de Coimbra, Azinhaga de Santa Comba, 3000-548 Coimbra, Portugal; ocardoso@ci.uc.pt; 2 CIMAGO (Centro de Investigação em Meio Ambiente, Genética e Oncobiologia), Faculdade de Medicina, Universidade de Coimbra, Azinhaga de Santa Comba, 3000-548 Coimbra, Portugal; mmdonato@fmed.uc.pt; 3 Research Institute for Medicines (iMed.ULisboa), Faculty of Pharmacy, Universidade de Lisboa, 1649-003 Lisboa, Portugal; sarachenriques@campus.ul.pt; 4 Universidade de Coimbra, Faculdade de Farmácia, REQUIMTE/LAQV, Azinhaga de Santa Comba, 3000-548 Coimbra, Portugal

**Keywords:** fluoroquinolone residues, *Escherichia coli*, *Enterococcus* species, antimicrobial resistance, piglets, One Health

## Abstract

The presence of fluoroquinolone (FQ) residues in food-producing animals has raised concerns regarding antimicrobial resistance. This study evaluated the occurrence of FQ residues in the liver and kidneys of piglets and their association with resistance patterns in *Escherichia coli* and *Enterococcus* species from piglets’ intestinal microbiota. FQ residues were found in 44% of livers and 23% of kidneys. Among 340 *E. coli* isolates from feces, resistance to ciprofloxacin (CIP) (43.2%) and multidrug resistance (MDR) (82.7%) were prominent. The presence of FQ in kidneys significantly increased the odds of CIP-resistant *E. coli* (OR = 2.94, *p* = 0.0013) and MDR (OR = 2.70, *p* = 0.0047). Enterococci (*n* = 130) were evenly distributed among the species *E. faecalis*, *E. faecium*, and *Enterococcus* spp. and presented resistance to CIP (34.6%). FQ in kidneys were associated with higher odds of CIP-resistant enterococci (OR = 3.78, *p* = 0.015). Interaction models revealed species-dependent responses, with *Enterococcus* spp. showing high resistance in the presence of FQ in kidneys (OR = 18, *p* = 0.006), while *E. faecium* exhibited lower resistance compared to *E. faecalis*. These findings emphasize the role of FQ residues in promoting bacterial resistance and underscore the need for the stricter regulation and routine surveillance of antimicrobial use in livestock to curb the spread of bacterial resistance to clinical drugs, and mitigate public health risks—One Health.

## 1. Introduction

The use of antibiotics has been of such an order of magnitude since their discovery by Fleming in 1921 that bacteria have organized themselves to avoid this stress inflicted on them. The World Health Organization (WHO) considers antibiotic resistance a global plague [1]. These resistant bacteria can appear anywhere, such as soil, water, animals, and plants (the One Health concept is very evident in this context), and when they reach humans by any means, they can cause serious illnesses, prolonged hospitalizations, etc. Antibiotics have been widely used worldwide, especially in modern animal husbandry, where they are often used as veterinary drugs for treating infectious diseases, like sulfonamides, quinolones, and macrolides [2]. Some antibiotics are even utilized as growth promoters, like small sub-therapeutic doses of tetracycline, to improve the animal’s immunity and increase production. However, the overconsumption of these veterinary antibiotics promotes the occurrence of antibiotic resistance in food animals and accelerates the dissemination process of antibiotic resistance [2].

Pig farming systems subject animals to significant stress, leading to various physiological disturbances, particularly in the gastrointestinal tract of piglets. Postweaning diarrhea is one of the most common and economically damaging conditions in pig farming. Antibiotics have been extensively used in pig production for decades to mitigate these adverse effects. However, the widespread use of antibiotics contributed to the emergence of resistant bacterial strains, raising serious public health concerns. In response, the European Union banned the inclusion of antibiotics in livestock feed in 2003.

The WHO has classified certain antimicrobial classes as critically important for human medicine, like fluoroquinolones. Despite their significance in human healthcare, these antibiotics are widely used in veterinary medicine, including swine farming, where enrofloxacin is a common choice. However, the fluoroquinolones used in animals can lead to the selection of resistance determinants to this class of antimicrobials, potentially compromising the effectiveness of ciprofloxacin in human therapy [3].

Once administered, fluoroquinolones exhibit rapid absorption with wide tissue dissemination and are excreted through urine and bile. Higher concentrations of drug residues are usually found in the liver and kidney, considering that the hepatobiliary system and the kidneys are the main routes by which drugs and their metabolites leave the body. Due to fluoroquinolones’ lipophilic characteristics, they have a long half-life and slow metabolization, increasing the risk of drug residues in animal-derived food products, which can pose health hazards to consumers [4].

A systematic review of fluoroquinolone resistance in isolates collected from healthy pigs showed that resistance to this group of antibiotics was variable in Europe. The highest resistance rates were observed in Portugal, Spain, Romania, and North Macedonia. The use of fluoroquinolones in pig farming has been banned in some countries; however, the persistence of high resistance levels in these regions indicates a strong correlation between antibiotic use in food animals and the development of resistance [3]. These findings highlight the ongoing challenges of antimicrobial resistance in animal production and reinforce the need for stringent monitoring and control measures to mitigate its spread.

*Escherichia coli* and the genus *Enterococcus* are commonly used as indicator organisms in many antimicrobial resistance surveillance programs [5]. *E. coli* and enterococci are ubiquitous and found in various environments, including the gastrointestinal tract, water, soil, and food. These properties make them valuable sentinel organisms for monitoring antimicrobial resistance trends and environmental transmission. Hence, surveillance programs that track the presence and patterns of antimicrobial resistance in *E. coli* and enterococci could provide the development of combating strategies for these global health threats [6,7,8].

This study aimed to detect fluoroquinolone (FQ) residues in the livers and kidneys of piglets collected in a Portuguese abattoir and assess whether bacterial representatives of their intestinal microbiota—such as *E. coli* and *Enterococcus* species—exhibit resistance to antibiotics used in human medicine and determine if there is a relationship between FQ residues in viscera and bacterial ciprofloxacin (CIP) resistance. Given the potential for these resistant bacteria to enter the environment and impact human health, the results of this study will be examined through the lens of the “One Health” approach.

## 2. Materials and Methods

### 2.1. Sampling and Escherichia coli and Enterococcus spp. Isolation

In this study, feces, liver, and kidney samples were collected from randomly selected healthy piglets (weighing 5–8 kg) between October 2018 and May 2019. The piglets came from an abattoir in Mealhada, which receives piglets from different regions of Portugal due to the high demand for roasting piglets (‘leitão à bairrada’), a local gastronomic specialty [9]. Sampling was carried out under a veterinarian’s supervision.

The samples collected from each animal consisted of one whole kidney; 200 g of the liver (right lobe); and a minimum of 10 g of feces. All samples were individually placed in sterile plastic bags and immediately transported to the laboratory. Liver and kidney samples were stored at −18 °C until analysis.

The isolation of *E. coli* was performed on the day of collection as described elsewhere using Lauryl Sulphate Agar [9], incubated at 37 °C/18 h, with the yellow-colored colonies on the agar being *E. coli*. Any doubts regarding identification were resolved with API20E (BioMérieux, Marcy-l-Étoile, France). In 2019, given the results obtained with Gram-negative bacteria, enterococci (Gram-positive bacteria) were also isolated to extend the study to another bacteria representative of the piglet microbiota. The isolation of *Enterococcus* species in the feces samples was performed [10] utilizing Slanetz Bartley Agar (Oxoid, Basingstoke, UK) and incubation at 37 °C, for 24–48 h. The red-colored colonies were representative of *Enterococcus* and for further identification, catalase, Bile Esculin Agar (Oxoid), and Trypticase Soy broth+6.5% NaCl tests were performed. If there was any doubt about the identification, API STREP (BioMérieux, France) was used.

### 2.2. Identification of Enterococcus Species

Real-time PCR (LightCycler, Roche Diagnostics, Germany) on crude bacterial DNA identified *Enterococcus* spp., *E. faecalis*, and *E. faecium* (Table 1).

PCR was performed in a volume of 20 µL containing 4.0 µL of LightCycler FastStart DNA MasterPLUS SYBR Green I^®^ (Roche Diagnostics, Mannheim, Germany). An initial denaturation cycle at 95 °C for 10 min was performed in all cases followed by 45 amplification cycles. Specific denaturation, annealing, and extension conditions for each set of primers are shown in Table 1. Melting curves were also plotted automatically and analyzed with the LightCycler software version 4.1. PCR products were checked on 2% agarose gels stained with ethidium bromide and visualized with UV light. In comparing both results, it was possible to establish specific melting temperatures to identify each gene, and positive and negative controls were always included. To avoid methodology errors, a different technician, blinded to previous results, repeated all procedures.

### 2.3. Antimicrobial Susceptibility Assays

The isolates were tested for antimicrobial susceptibility using the agar disk diffusion method on Mueller–Hinton agar according to the guidelines provided by the European Committee for Antimicrobial Susceptibility Testing [12].

For the antibiogram of *E. coli* isolates, the following antimicrobial agents (Liofilchem^®^s.r.l., Roseto degli Abruzzi TE, Italy) were used and included the following four families of beta-lactams: amoxicillin–clavulanic acid (AMC) (20–10 µg); piperacillin (PIP) (30 µg); cefoxitin (FOX) (30 µg); ceftazidime (CAZ) (10 µg); cefepime (FEP) (30 µg); imipenem (IP) (10 µg); and aztreonam (AZT) (30 µg). The remaining 3 antimicrobials were from other families and included sulfamethoxazole-trimethoprim (SXT) (23.75–1.25 µg); ciprofloxacin (CIP) (5 µg); and amikacin (AK) (30 µg). *E. coli* ATCC 25922 was used as a control.

In the antibiogram of *Enterococcus* species, the antimicrobials were vancomycin (VAN), ciprofloxacin (CIP), linezolid (LNZ), tigecycline (TIG), ampicillin (AMP), and imipenem (IP). *E. faecalis* ATCC 29212 was used as a control.

### 2.4. Quantification of Antimicrobial Residues

UHPLC-ToF-MS was used to determine the six fluoroquinolones (FQs) (enrofloxacin, ciprofloxacin, danofloxacin, ofloxacin, norfloxacin, and marbofloxacin) in the liver and kidney. Antimicrobial and internal standards of ≥98% purity were purchased from Sigma-Aldrich (Madrid, Spain). The extraction procedure and UHPLC-ToF-MS conditions have been described previously [13].

### 2.5. Statistical Analysis

Data are presented as absolute and/or relative frequencies. Associations were evaluated using logistic regression models, with a significance level of 5%. Analyses were performed in R software version 4.3.2 (R Foundation for Statistical Computing, Vienna, Austria).

## 3. Results

### 3.1. Occurrence of Fluoroquinolone Residues in Piglets’ Liver and Kidney

In 2018, 32 liver and kidney samples were collected from piglets, of which 13 livers and 7 kidneys presented FQs. In 2019, 43 samples were obtained, and 20 livers and 10 kidneys presented FQs. In total, 44% of the livers and 23% of the kidneys had FQ residues. Piglets in which FQ was detected in the kidney also had FQ detected in the liver (*n* = 17). In total, 75 piglets were analyzed: 33 presented FQ residues in the liver (44%), and 17 in the kidney (23%).

### 3.2. Escherichia coli and Antibiotic Resistance

A total of 340 *E. coli* isolates were identified from 75 piglets’ feces. Table 2 presents the bacteria’s resistance profiles to antibiotics used in human therapeutics.

Higher resistance was observed for penicillins (AMC and PIP), followed by SXT and CIP. A total of 321 (82.7%) isolates exhibited resistance to two or more antibiotics, while 208 (61.2%) isolates were resistant to three or more antibiotics, classifying them as multidrug-resistant (MDR) bacteria.

### 3.3. Association Between FQ Residues in Viscera and E. coli CIP Resistance

In the 33 piglets with FQ residues in the liver, 159 *E. coli* isolates were identified, of which 94 (59.1%) exhibited resistance to CIP, while 65 (40.9%) were susceptible. When FQ was absent from the liver, 181 *E. coli* isolates were identified, with 53 (29.3%) showing resistance to CIP and 128 (70.7%) being susceptible.

**Figure 1:**
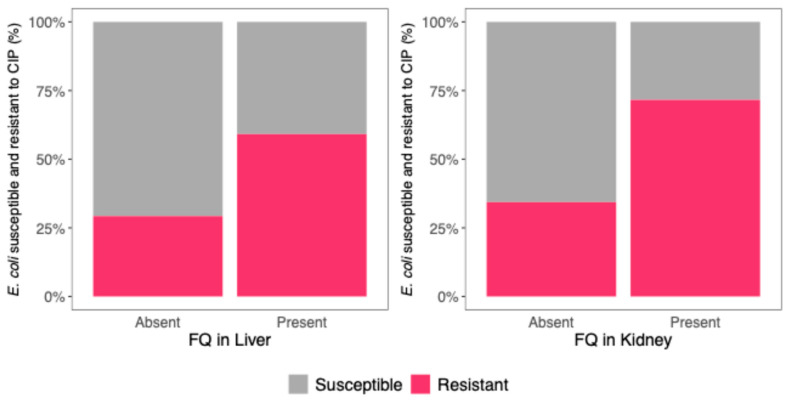
*E. coli* isolates (%) susceptible (gray) and resistant (pink) to CIP, as a function of the presence or absence of FQ residues in the liver (**left**) and kidney (**right**).

Among the 17 piglets in which FQ residues were detected in the kidney, 81 *E. coli* isolates were identified. Of these, 58 (71.6%) were resistant to CIP, while 23 (28.4%) were susceptible. In contrast, when FQ was absent from the kidney, 259 *E. coli* isolates were identified, of which 89 (34.4%) were resistant to CIP and 170 (65.6%) were susceptible (Figure 1).

A mixed effects logistic regression model was used to assess the relationship between FQ residues in piglet organs and *E. coli* resistance to CIP. The final model included the FQ residues in the liver and kidney as predictors, with the piglet from which the residues were taken used as a random effect to account for the clustering of isolates within the animals.

The model showed that the presence of FQ residues in the kidney was associated with a 2.94-fold increase in the odds of *E. coli* CIP resistance (odds ratio [OR] 95% CI: 1.57–5.50, *p* = 0.0013). Likewise, FQ residues in the liver were associated with a 2.07-fold increase in the odds of *E. coli* CIP resistance (OR 95% CI: 1.20–3.57, *p* = 0.0093) (Figure 1, Table 3).

These results suggest that FQ residues in the liver and kidney contribute to developing or maintaining CIP resistance in *E. coli*.

### 3.4. Association Between FQ Residues and Multidrug Resistance in E. coli

The relationship between FQ residues in piglet organs and MDR in *E. coli* isolates was also evaluated.

**Figure 2:**
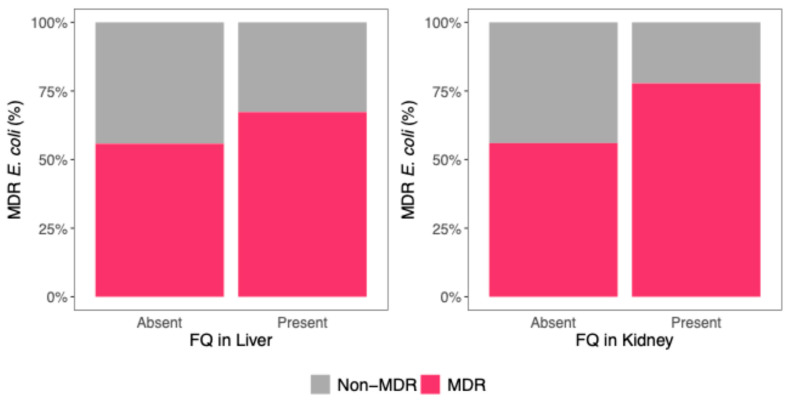
MDR *E. coli* (%) (pink) as a function of the presence or absence of FQ residues in the liver (**left**) and kidney (**right**).

In piglets with FQ residues in the liver, 159 *E. coli* isolates were identified, of which 107 (67.3%) were MDR, while 52 (32.7%) were non-MDR. When FQs were absent from the liver, 181 isolates were identified: 101 (55.8%) MDR and 80 (44.2%) non-MDR (Figure 2 left).

Among the 17 piglets with FQ residues in the kidney, 81 *E. coli* isolates were identified. Of these, 63 (77.8%) were MDR, while 18 (22.2%) were non-MDR. In contrast, when FQ was absent from the kidney, 259 isolates were identified, of which 145 (56.0%) were MDR and 114 (44.0%) were non-MDR (Figure 2 right).

A logistic regression model was fitted to evaluate the association between FQ residues in the liver and kidney and the MDR *E. coli*. The presence of FQ in the kidney was significantly associated with a 2.70-fold increase in the likelihood of MDR (OR 95% CI: 1.37–5.47, *p* = 0.0047). However, FQ residues in the liver were not significantly associated with MDR (OR: 1.03, 95% CI: 0.60–1.76, *p* = 0.93) (Figure 2, Table 3).

### 3.5. Enterococcus Species Identification and Antibiotic Resistance

From 43 piglet feces, a total of 130 enterococci isolates were identified and distributed among the Enterococci species as follows: 40 (30.8%) *Enterococcus faecalis*; 45 (34.6%) *Enterococcus faecium*; and 45 (34.6%) *Enterococcus* spp. The distribution was relatively even, with each species represented in similar proportions. The estimated Shannon diversity index of 1.097, close to the maximum possible value of *ln*(3) = 1.0986, indicates a diverse and balanced community, with no dominant species.

Table 4 presents the resistance profiles of enterococci to the different antibiotics utilized in human clinical therapeutic strategies. These species showed the highest resistance to CIP and IP, while VAN, LNZ, and AMP showed much lower levels of resistance and no resistance to TIG.

### 3.6. Association Between FQ Residues in Viscera and Enterococci CIP Resistance

In the 20 piglets with FQ residues in the liver, 67 enterococci isolates were identified, with 25 (37.3%) showing resistance to CIP and 42 (62.7%) being susceptible. When FQ residues were absent from the liver, 63 isolates were identified, of which 20 (31.7%) were resistant and 43 (68.3%) were susceptible.

Among the 10 piglets in which FQ residues were detected in the kidney, 35 enterococci isolates were identified. Of these, 18 (51.4%) were resistant to CIP, while 17 (48.6%) were susceptible. In contrast, when FQ residues were absent from the kidney, 95 isolates were identified, of which 27 (28.4%) were resistant and 68 (71.6%) were susceptible.

A mixed effects logistic regression model was used to evaluate the association between the presence of FQ residues in piglet organs and bacterial resistance to CIP. The model included the presence of FQs in the liver and kidney as predictors, with piglet identification as a random effect.

**Figure 3:**
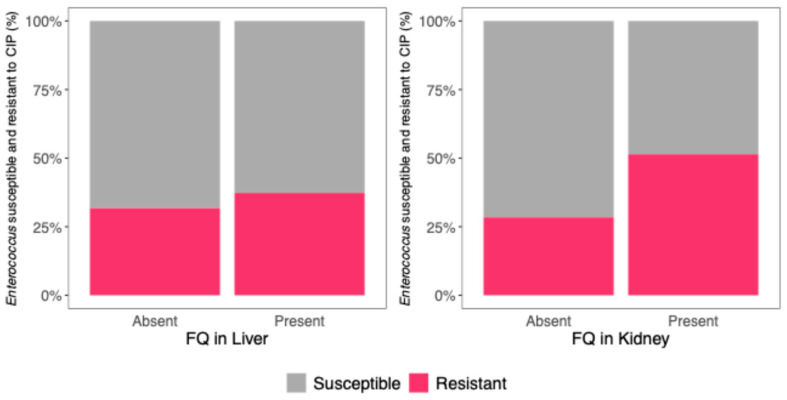
*Enterococcus* species isolates (%) susceptible (gray) and resistant (pink) to CIP, as a function of the presence or absence of FQ residues in the liver (**left**) and kidney (**right**).

The model showed that the presence of FQ residue in the kidney was significantly associated with a 3.78-fold increase in the odds of enterococci CIP resistance (OR 95% CI: 1.34–11.60, *p* = 0.015). In contrast, FQ residues in the liver were not significantly associated with enterococci CIP resistance (OR = 0.60, 95% CI: 0.21–1.57, *p* = 0.316) (Figure 3, Table 3).

These results suggest that FQ residues in the kidney played a critical role in developing and maintaining CIP resistance in enterococci.

Moreover, species-specific resistance patterns were also observed for CIP: for *E. faecalis*, 18 isolates (45.0%) were resistant to CIP, while 22 (55.0%) were susceptible. For *E. faecium*, 7 isolates (15.6%) were resistant, whereas 38 (84.4%) were susceptible. For *Enterococcus* spp., 20 isolates (44.4%) were resistant, and 25 (55.6%) were susceptible.

Among the 35 enterococci isolates from piglets with FQ residues detected in the kidney, 18 (51.4%) were resistant to CIP, while 17 (48.6%) were susceptible: *E. faecalis*: 4 resistant, 7 susceptible; *E. faecium:* 2 resistant, 7 susceptible; *Enterococcus* spp.: 12 resistant, 3 susceptible.

Among the 95 enterococci isolates from piglets where FQ residues were not detected in the kidney, 27 (28.4%) were resistant to CIP, while 68 (71.6%) were susceptible: *E. faecalis*: 14 resistant, 15 susceptible; *E. faecium:* 5 resistant, 31 susceptible; *Enterococcus* spp.: 8 resistant, 22 susceptible.

To assess whether CIP resistance varied among *Enterococcus* species, a new logistic regression model was fitted, incorporating an interaction term between FQ presence in the kidney and *Enterococcus* species. This model showed that *E. faecium* had significantly lower odds of resistance compared to *E. faecalis* (OR = 0.17, 95% CI: 0.05–0.54, *p* = 0.004). No significant difference was observed between *Enterococcus* spp. and *E. faecalis* in terms of CIP resistance (OR = 0.39, 95% CI: 0.13–1.14, *p* = 0.090). However, the presence of FQ residues in the kidney significantly increased CIP resistance in *Enterococcus* spp. The interaction term showed that when FQ residues were present in the kidney, *Enterococcus* spp. had an approximately 18-fold higher likelihood of CIP resistance compared to *E. faecalis* without FQ residues in the kidney (OR 95% CI: 2.43–160.56, *p* = 0.006).

These results suggest that FQ residues in the kidney enhance CIP resistance in certain *Enterococcus* species, particularly *Enterococcus* spp.

Among the 67 enterococci isolates from piglets with FQ residues in the liver, 25 (37.3%) were resistant to ciprofloxacin, while 42 (62.7%) were susceptible: *E. faecalis*: 6 resistant, 11 susceptible; *E. faecium:* 2 resistant, 17 susceptible; *Enterococcus* spp.: 17 resistant, 14 susceptible.

Among the 63 enterococci isolates from piglets where FQ residues were not detected in the liver, 20 (31.7%) were resistant to CIP, while 43 (68.3%) were susceptible: *E. faecalis*: 12 resistant, 11 susceptible; *E. faecium*: 5 resistant, 21 susceptible; *Enterococcus* spp: 3 resistant, 11 susceptible.

A logistic regression model was used to evaluate the association between FQ residues in the liver and CIP resistance, including an interaction term for *Enterococcus* species. This model showed that *E. faecium* had significantly lower odds of resistance compared to *E. faecalis* (OR = 0.22, 95% CI: 0.06–0.75, *p* = 0.019). No significant difference was observed between *Enterococcus* spp. and *E. faecalis* in terms of CIP resistance (OR = 0.25, 95% CI: 0.05–1.05, *p* = 0.073).

However, the interaction term showed that when FQ residues were present in the liver, *Enterococcus* spp. had an approximately nine-fold higher likelihood of CIP resistance compared to *E. faecalis* without FQ residues (OR 95% CI: 1.35–69.51, *p* = 0.028). These results suggest that FQ residues in the liver had a species-dependent impact on enterococci CIP resistance, particularly in increasing in *Enterococcus* spp.

## 4. Discussion

This study highlights the presence of FQ residues in piglet livers and kidneys and their association with antimicrobial resistance in *E. coli* and enterococci. These findings have important implications for antibiotic stewardship in livestock production and the potential risks of antimicrobial resistance transmission to humans [3,14].

FQ residues in piglet liver and kidney were high, with 44% of livers and 23% of kidneys containing detectable levels. These results are consistent with previous studies demonstrating the persistence of FQ in animal tissues [4,15]. The detection of these residues indicates significant exposure of piglets to FQ [16]. The co-occurrence of FQ residues in both liver and kidney in 17 piglets suggests systemic exposure, possibly due to prolonged administration or inadequate withdrawal periods before slaughter [4,14,17].

This study also suggests that the presence of FQ residues in piglet organs is associated with increased resistance to CIP in both *E. coli* and *Enterococcus* species, raising concerns about the role of antibiotic residues in promoting antimicrobial resistance [18,19].

The observed association between FQ residues and CIP-resistant *E. coli* was of particular concern. The logistic regression model confirmed that the presence of FQ residues in the kidney significantly increased the odds of CIP resistance (OR = 2.94, *p* = 0.0013), with a slightly lower but still significant effect in the liver (OR = 2.07, *p* = 0.0093). These data were consistent with other studies [18]. The results highlight the selective pressure exerted by FQ residues that might drive the emergence and persistence of resistant bacterial strains [3,20,21]. This study also demonstrated an association between FQ residues and MDR *E. coli*. FQ residues in the kidney were significantly associated with MDR *E. coli* (OR = 2.70, *p* = 0.0047), but the effect was insignificant for residues in the liver (OR = 1.03, *p* = 0.93). These results are consistent with previous findings in [22], which reported that FQ exposure in livestock contributes to the emergence of MDR strains. The higher MDR rate in kidney-associated *E. coli* isolates suggests that FQ residues may exert more selective pressure in this organ, possibly due to higher drug concentrations in renal tissue [23].

This study further evaluated the relationship between FQ residues and CIP resistance in *Enterococcus* species. While FQ residues in the kidney significantly increased the odds of enterococci CIP resistance (OR = 3.93, *p* = 0.016), the presence of FQ in the liver was not significantly associated with this resistance (OR = 0.50, *p* = 0.196). This suggests that renal accumulation of FQ might have a greater impact on enterococcal resistance selection [23].

The identification of 130 enterococcal isolates with a balanced species distribution indicates a diverse microbial population in piglet feces [24]. Enterococcal species showed different levels of CIP resistance, with *E. faecium* showing significantly lower odds of resistance compared to other species (OR = 0.24, *p* = 0.006); these results parallel the findings of [25,26]. *E. faecium* isolates showed high resistance to imipenem, a carbapenem of hospital use, but this species has an intrinsic tolerance to beta-lactams due to the presence of PBPs (specifically PBP5) with low binding affinity for this class of antibiotics [27]. All the enterococcus were susceptible to tigecycline, as stated by other authors [28].

The results of this study underscore the risks posed by antibiotic residues in livestock production. The significant associations between FQ residues and antibiotic-resistant bacteria reinforce the need for strict regulations on the use of antibiotics in food-producing animals. The European Food Safety Authority (EFSA) and the World Health Organization (WHO) have previously emphasized the threat of antimicrobial resistance posed by antibiotic residues in food of animal origin [5,16]. These findings reinforce these concerns and suggest that monitoring and limiting the use of FQs in piglets could help reduce the spread of bacterial resistance.

## 5. Conclusions

This study demonstrates that FQ residues in piglet livers and kidneys are significantly associated with increased resistance to CIP in *E. coli* and *Enterococcus* species. The presence of FQ residues in the kidney was particularly influential, considerably improving the odds of CIP resistance and MDR in *E. coli*. These findings highlight the urgent need for antibiotic stewardship in livestock production to reduce the risk of antimicrobial resistance transmission to humans in line with the One Health concept.

## Figures and Tables

**Table 1:** Primers and PCR conditions used in the identification of *Enterococcus* species.

Species	Primer Sequence	PCR Conditions	Amplicon Size (bp)	Reference
*Enterococcus* spp.	TCA ACC GGG GAG GGT	95 °C, 10 s; 60 °C, 5 s; 72 °C, 29 s	733	[11]
	ATT ACT AGC GAT TCC GG			
*E. faecalis*	ATC AAG TAC AGT TAG TCT	95 °C, 10 s; 44 °C, 5 s; 72 °C, 38 s	941	[11]
	ACG ATT CAA AGC TAA CTG			
*E. faecium*	TTG AGG CAG ACC AGA TTG ACG	95 °C, 10 s; 55 °C, 5 s; 72 °C, 26 s	658	[11]
	TAT GAC AGC GAC TCC GAT TCC			

**Table 2:** Number (%) of *E. coli* isolates with resistance to antibiotics.

Antibiotic	Total
Amikacin (AMK)	115 (33.8%)
Ciprofloxacin (CIP)	147 (43.2%)
Sulfamethoxazole-Trimethoprim (SXT)	197 (57.9%)
Aztreonam (AZT)	64 (18.8%)
Cefoxitin (FOX)	42 (12.4%)
Cefepime (FEP)	59 (17.4%)
Ceftazidime (CAZ)	39 (11.5%)
Amoxicillin + Clavulanic Acid (AMC)	228 (67.1%)
Piperacillin (PIP)	229 (67.4%)
Imipenem (IP)	9 (2.6%)

**Table 3:** Odds ratio in the relationship between FQ residues in piglet organs and bacterial CIP resistance.

	FQ Residues	
	Kidney OR (95% CI), *p*-Value	Liver OR (95% CI, *p*-Value)
*E. coli* resistance to CIP	2.94 (1.57–5.50), *p* = 0.0013	2.07 (1.20–3.57), *p* = 0.0093
MDR *E. coli*	2.70 (1.37–5.47), *p* = 0.0047	1.03 (0.60–1.76), *p* = 0.930
*Enterococcus* resistance to CIP	3.78 (1.34–11.60), *p* = 0.015	0.60 (0.21–1.57), *p* = 0.316

FQ—fluoroquinolone; CIP—ciprofloxacin; MDR—multi-drug resistance; OR—odds ratio; CI—confidence interval.

**Table 4:** Number of enterococci isolates with resistance to antibiotics.

Antibiotic	*E. faecalis* (*n* = 40)	*E. faecium* (*n* = 45)	*Enterococcus* spp. (*n* = 45)	Total (*n* = 130)
Vancomycin (VAN)	1	1	3	5
Ciprofloxacin (CIP)	18	7	20	45
Linezolid (LNZ)	0	1	1	2
Tigecycline (TIG)	0	0	0	0
Ampicillin (AMP)	1	2	1	4
Imipenem (IP)	6	29	6	41

## Data Availability

No new data were created or analyzed in this study. Data sharing is not applicable to this article.
